# Five interdisciplinary tensions and opportunities in neurodiversity research

**DOI:** 10.7554/eLife.98461

**Published:** 2024-04-23

**Authors:** Olujolagbe Layinka, Luca D Hargitai, Punit Shah, Lucy H Waldren, Florence YN Leung

**Affiliations:** 1 Department of Psychology, University of Bath Bath United Kingdom

**Keywords:** point of view, neurodiversity, interdisciplinary research, categorial disorders, dimensional traits, Human

## Abstract

Improving our understanding of autism, ADHD, dyslexia and other neurodevelopmental conditions requires collaborations between genetics, psychiatry, the social sciences and other fields of research.

## Introduction

Neurodiversity celebrates the inherent complexity of the human mind, highlighting the natural variations that are found within the population. Its higher profile in recent years has led to a greater awareness and more frequent diagnoses of neurodevelopmental conditions, such as autism, attention deficit hyperactivity disorder (ADHD), dyslexia, dyscalculia, dyspraxia and beyond.

Our understanding of these conditions is shaped by findings from a range of research disciplines, including the developmental and social sciences, cognitive neuroscience, psychiatry, education, and the clinical, health and biomedical sciences (notably neurobiology and genetics). Together, these fields aim to uncover the mechanisms and markers underlying neurodevelopmental conditions, from genetic to societal levels of explanation.

However, with so much heterogeneity within neurodevelopmental conditions and across these research domains, it is not surprising that there is a striking dearth of truly interdisciplinary neurodiversity research. This shortfall underscores the overlooked opportunities for the synergy that would come with such work. This article explores five key tensions in the field, and signposts potential opportunities for future progress.

## 1. Dimensional traits versus categorical disorders

Traditionally, researchers have viewed neurodevelopmental conditions as discrete, categorical ‘disorders’, in line with the *Diagnostic and Statistical Manual of Mental Disorders* (American Psychiatric Association, 2013). This has naturally facilitated the medicalisation of neurodevelopmental conditions and the view that they stem from biological deficits rooted within an individual.

Recently, however, researchers have argued that neurodevelopmental conditions, especially autism, are better conceptualised as a dimensional spectrum of measurable characteristics – or traits – that are continuously distributed across the general population (e.g., Happé and Frith, 2020). This ‘trait approach’ – unlike the disorder approach – fundamentally aligns with the inclusive ethos of the neurodiversity movement, with neurodivergent traits considered part of the natural variation within the population.

Some argue that a dimensional view of neurodevelopmental conditions perpetuates unhelpful myths that anyone can be a ‘bit autistic’ or a ‘bit dyspraxic’. For example, it is widely debated whether autistic traits are qualitatively and quantitatively distinct from an autism spectrum disorder diagnosis (Happé and Frith, 2020). However, the evidence now clearly indicates that sub-threshold diagnoses of neurodevelopmental conditions (i.e., high levels of traits that do not reach clinical diagnostic thresholds) are both common and predictive of poor health outcomes (Thapar et al., 2017). The trait approach is also better suited to explain the complex variability in characteristics among people with the same diagnosis (Lyall, 2023). Moreover, the trait approach is more closely linked to the biological processes underlying neurodevelopmental conditions (e.g., certain genes are linked to specific autistic traits, rather than to an autism diagnosis; Lee et al., 2022).

It is, therefore, time for research to embrace neurodiversity and leverage the trait approach to study the shared and distinct mechanisms underlying neurodevelopmental conditions. This can be accomplished through conducting transdiagnostic research under the Research Domain Criteria framework, which explores a range of trait domains (e.g., sensorimotor, regulatory, cognitive, social processes) across multiple levels of explanation (e.g., behavioural, genetic, physiological perspectives; see Cuthbert and Insel, 2013). If utilised successfully, such a framework could lead to a far more nuanced understanding of individual differences both within and beyond classical diagnostic ‘disorder’ boundaries.

## 2. Your word against mine

Given the significant shift in our understanding towards the neurodiversity paradigm, it is unsurprising that we have also seen a sizable shift in our terminology. Whilst research has traditionally used deficit-based language, the neurodiverse community has called for a more inclusive approach (Monk et al., 2022). However, such terminology lends itself better to some disciplines than others. For example, inclusive language is more commonly used within behavioural and cognitive research, and the journal *Neurodiversity* (which is co-edited by PS) actively discourages deficit-based language. In contrast, biomedically grounded disciplines, by their very nature, are more steadfast in their use of standardised medical parlance. Such discrepancies have created heated tensions between different disciplines, and between the neurodiverse community and certain researchers. Consequently, trust needs to be established if interdisciplinary neurodiversity research hopes to succeed.

Not only is there a discrepancy in the use of language between disciplines, but also in fundamental definitions that are pertinent to neurodiversity research. Many overlapping but distinct terms are used to describe the same constructs in the literature, making it difficult to untangle the specific mechanisms and processes being observed. For example, ‘sensitivity’, ‘reactivity’ and ‘responsivity’ have all been used interchangeably to describe sensory processing differences in various neurodevelopmental conditions (see He et al., 2023 for a discussion). Such inconsistencies in terminology reflect the ‘jingle jangle fallacy’ (the misleading assumptions that two different things are the same because they bear the same name, or that two identical things are different because they have a different name) that is increasingly prevalent in scientific research as the literature expands rapidly across different disciplines. This confusion represents a serious barrier to developing an interdisciplinary understanding of neurodevelopmental processes. To stand any chance of harmonising research findings across disparate disciplines, researchers first need to agree on standardised terminology – or, at the very least, clear definitions – of the neurodevelopmental processes they wish to investigate (Shah et al., 2022).

## 3. Finding a common goal

Such discrepancy in terminology feeds into an even more fundamental challenge facing neurodiversity research: the inconsistency in how it is investigated between disciplines. Within the genetic and neuroscience fields, research has focused on the medicalisation of neurodevelopmental conditions and finding potential ‘targets’ for personalised interventions at a neurobiological level. Indeed, hundreds of genes have been associated with every neurodevelopmental condition (e.g., Genovese and Butler, 2023). However, paradoxically, whilst these disciplines contribute the ‘neuro’ to neurodiversity, there is increasing misalignment between their goals and those of the people they aim to help (Russell and Wilkinson, 2023). For example, studies have found that members of the autistic community want to see research focused on topics that will have a tangible impact and lead to improvements in their day-to-day lives, rather than on potential biological mechanisms that may underpin certain ‘deficits’ (Pellicano et al., 2014).

Fortunately, opportunities for interdisciplinary discussions between academics and neurodiverse communities are now emerging, especially in the UK and US (such as the Autistica Research Festival, the International Society for Autism Research Annual Meetings and the It Takes All Kinds Of Minds Conference), and we encourage the wider adoption of such events internationally. These highly attended platforms offer a respectful and stimulating space for mutual collaborative learning that helps to align research goals. In addition, public engagement activities that enhance the translational impact of research findings create a real potential to mend the researcher-community relationships that are currently fractured.

## 4. Resource allocation

One barrier to achieving these mutual goals is the disproportionate allocation of resource funding across different disciplines. For example, between 2014 and 2017, 47% of mental health research funding in the UK went into deficit-focused, biomedically grounded disciplines, while only 9% of research funding was allocated to improving mental health services (MQ Transforming Mental Health, 2021).

**Figure 1.**
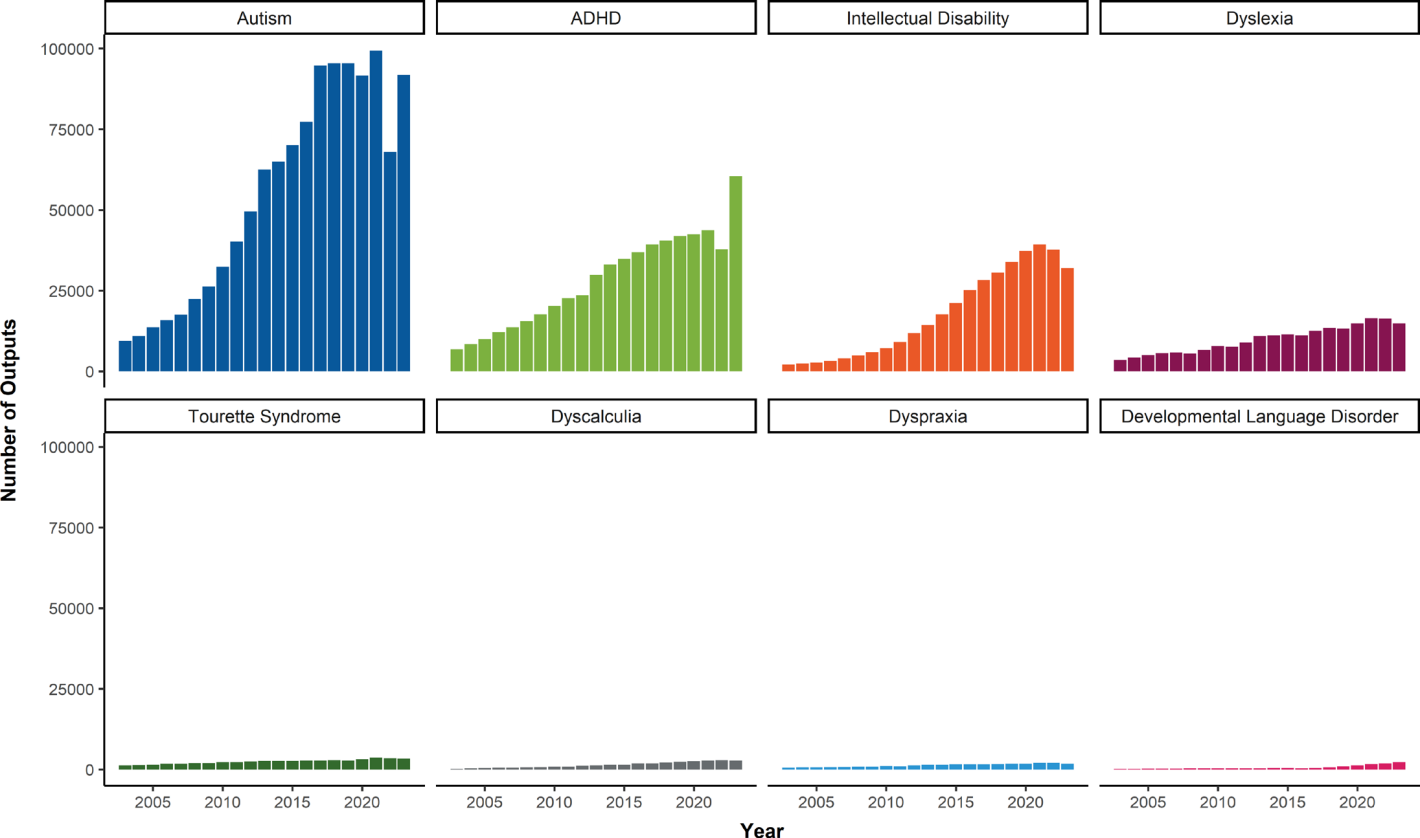
Number of scholarly outputs versus year for eight neurodevelopmental conditions between 2003 and 2023. Data extracted from Google Scholar; the eight conditions are ordered by number of scholarly outputs.

Moreover, comparing funding for different neurodevelopmental conditions reveals that the amount of funding for research into autism in the UK is comparable with the funding available for all other neurodevelopmental conditions combined. This evident ‘favouritism’ amongst funding bodies leads to a vicious funding-research cycle that leaves lesser-known conditions behind, such as dyscalculia and dyspraxia (Figure 1; Bishop, 2010).

Yet, neurodiversity research would gain immensely from collaboration between the different research fields. For example, data obtained from brain imaging and eye-tracking measures could complement the interpretation of behavioural performance on cognitive tasks. This, in turn, could tell us something meaningful about the (compensatory) processing strategies employed across different neurodevelopmental conditions, such as autism and dyspraxia (Livingston and Happé, 2017). This could relieve some of the financial restraints associated with under-funded conditions and disciplines, while maximising our insights on the complexities and links between neurodevelopmental conditions.

## 5. Balancing advocacy with accuracy

Beyond research funding, a greater balance needs to be established in how we incorporate neurodivergent perspectives into research. In line with this, inclusive research practices (e.g., meaningful co-production, patient and public involvement opportunities, neurodivergent advisory boards) have been recommended to help achieve research excellence and ensure that research aligns with the priorities of the wider neurodiverse community (Hobson et al., 2023). However, this process is not straightforward, as contrasting voices add to existing tensions and some perspectives are disproportionately heard over others.

This issue is exemplified by the controversy surrounding the recent Spectrum 10K genome-wide association study in autism, which was suspended after concerns were raised by certain figures within the neurodiverse community (Natri, 2021). Despite this outcry, subsequent research found that parents of nonverbal autistic children actually support genetic research and feel that their children are frequently excluded and underrepresented by the autism community (Asbury et al., 2024). This sentiment is echoed by those considered less ‘high-functioning’ (Singer, 2022), and this is also reflected in research participant demographics (Russell et al., 2019). Together, these insights prompt consideration of the reliability and representativeness of commonly accepted perspectives from neurodivergent people, ensuring they accurately reflect the entire community.

It is, therefore, important that researchers consider whether the ‘experts by experience’ that are informing their research are indeed representative of the diverse individuals they wish to understand. Certain initiatives are already in place to rely less on patient and public involvement panels and to systematically obtain a broader range of perspectives. For example, the Regulating Emotions – Strengthening Adolescent Resilience (RE-STAR) programme has recently built a new participatory model, offering guidance on how to effectively integrate young people with ADHD and/or autism into translational research (Sonuga-Barke et al., 2024).

When paired with open science practices, these different approaches could prove invaluable for translating valid and reliable research findings into something impactful for the community (Hobson et al., 2023). For example, preregistering a research plan in advance of a study requires researchers to think more carefully about their research questions, study design and target populations, which can help to contextualise and interpret research findings. Equally, making data and analysis codes openly accessible allows reported findings to be independently verified by other researchers, raising the overall standard of neurodiversity research.

## Where does neurodiversity start and stop?

Whilst this article has covered key tensions and opportunities in neurodiversity research, we have drawn on research and ongoing real-world issues pertaining to ‘common’ neurodevelopmental conditions. This is a limitation, and it is important to acknowledge that neurodiversity is a very broad, ever-expanding umbrella term, which may continue to widen as additional conditions, such as schizophrenia, bipolar disorder and epilepsy are included by proponents of the neurodiversity concept (Morris-Rosendahl and Crocq, 2020). Indeed, given their links to cognitive and perceptual differences in neural development, conditions such as developmental prosopagnosia (a condition that makes it difficult to recognise faces), congenital amusia (tone deafness) and aphantasia (a condition that makes it difficult to create mental images) could also fall under this umbrella term.

An existential question that remains to be answered in neurodiversity research, therefore, concerns where the boundaries of neurodiversity lie. Understanding where neurodiversity starts and stops has the potential to tangibly shape research in this field, making it more empirically tractable. But should there even be a starting and stopping point if neurodiversity is to be considered a natural part of human variation? And, if not, do we ultimately have to dispense with the concept of conditions? We cannot be sure of the answer to this conundrum, but we are certain that tackling this fundamental question will only be achieved if researchers work collaboratively – across disciplines and together with the neurodiverse community – to produce high-quality research.
